# P-807. Evaluation of Risk Factors for Carbapenem-Discordant Enterobacterales Bacteremia at a Large Academic Cancer Institution

**DOI:** 10.1093/ofid/ofae631.999

**Published:** 2025-01-29

**Authors:** Alex Stabler, Nancy Vuong, Jovan Borjan, Hyunsoo Hwang, Chun Feng

**Affiliations:** University of Texas MD Anderson Cancer Center, Houston, Texas; University of Texas MD Anderson Cancer Center, Houston, Texas; The University of Texas MD Anderson Cancer Center, Houston, TX; The University of Texas MD Anderson Cancer Center, Houston, TX; The University of Texas MD Anderson Cancer Center, Houston, TX

## Abstract

**Background:**

Carbapenem resistance in non-carbapenemase producing Enterobacterales is thought to be mediated by increased beta-lactamase production and outer membrane porin mutations. Cases of phenotypic discordance of carbapenem susceptibility (ertapenem-nonsusceptible, meropenem-susceptible) are documented. Given limited clinical data in immunocompromised patients, we aim to characterize risk factors that may lead to carbapenem-discordant Enterobacterales (CD-E) bloodstream infection (BSI) in patients with cancer.

**Methods:**

This is a retrospective, single-center study comparing adults > 18 years of age from January 2016 through November 2023 with a history of a ceftriaxone-resistant Enterobacterales (CRO-R-E) BSI subsequently having either a CRO-R-E or CD-E BSI of the same index organism within one year. Fisher’s exact test was used to compare categorical variables. Wilcoxon rank-sum test was used for continuous variables. Univariate logistic regression models were used to evaluate risk factors associated with CD-E BSI development.

**Results:**

We evaluated 829 patients with CRO-R-E BSI. 9.4% had a subsequent CRO-R-E BSI and 1.6% developed CD-E BSI with the same index organism. The most common organisms identified were Escherichia coli (n=70, 76.9%) and Klebsiella species (n=16, 17.6%). There was no difference in age and most patients had a hematologic malignancy (n=83, 91.2%). Subsequent CRO-R-E or CD-E BSI occurred most often within 90 days of the index BSI (n=61, 78% and n=11, 85%, respectively). Ceftazidime-avibactam (CZA) and latter generation cephalosporin exposure were each significantly associated with CD-E BSI (OR 10.29; p< 0.001 and OR 1.05; p=0.03, respectively). Severe neutropenia (absolute neutrophil count < 500 K/µL) trended towards significance for CD-E BSI development (OR 3.41, p=0.06). Of the 13 CD-E BSI, development of nonsusceptible carbapenem-concordant phenotype with the same organism in the following year occurred in 5 patients (38.5%).

**Conclusion:**

These data suggest that risk factors associated with CD-E BSI development include exposure to CZA or latter generation cephalosporins. Severe neutropenia may also be an indicator, but further studies are warranted to validate this and other CD-E BSI risk factors.

**Disclosures:**

**All Authors**: No reported disclosures

